# Traffic and log data captured during a cyber defense exercise

**DOI:** 10.1016/j.dib.2020.105784

**Published:** 2020-05-29

**Authors:** Daniel Tovarňák, Stanislav Špaček, Jan Vykopal

**Affiliations:** a Institute of Computer Science, Masaryk University, Brno, Czech Republic; b Faculty of Informatics, Masaryk University, Brno, Czech Republic

**Keywords:** Cybersecurity, Cyber defense exercise, Network traffic, Network flow, Event log, Syslog, KYPO

## Abstract

Cybersecurity research relies on relevant datasets providing researchers a snapshot of network traffic generated by current users and modern applications and services. The lack of datasets coming from a realistic network environment leads to inefficiency of newly designed methods that are not useful in practice. This data article provides network traffic flows and event logs (Linux and Windows) from a two-day cyber defense exercise involving attackers, defenders, and fictitious users operating in a virtual *exercise network*. The data are stored as structured JSON, including data schemes and data dictionaries, ready for direct processing. Network topology of the *exercise network* in NetJSON format is also provided.

Specifications table

**Table:** 

**Subject**	Computer Networks and Communications
**Specific subject area**	cybersecurity, computer networks, intrusion detection, cybersecurity education
**Type of data**	Network topology description in NetJSON; IPFIX traffic flows in structured JSON; Linux Syslog data in structured JSON; Windows Event Log data in structured JSON; Table; Image; Figure
**How data were acquired**	The data were acquired during Cyber Czech – a hands-on cyber defense exercise (Red Team/Blue Team) held in March 2019 at Masaryk University, Brno, Czech Republic. Network traffic flows and a high variety of event logs were captured in an *exercise network* deployed in the KYPO Cyber Range Platform.
**Data format**	Raw
**Parameters for data collection**	All traffic passing through the observation point was captured and exported. The event logs were captured based on the individual host configurations. All *Blue Team networks* and hosts were configured identically at the start of the exercise. No traffic within the Blue/Red Team networks was captured. The network traffic and the event logs were captured exclusively in the *exercise network*.
**Description of data collection**	The network traffic was captured on a single network interface (observation point) in the *exercise network* and then exported into IPFIX flows. Through this observation point, traffic between each Blue Team and the *global network* passed through. The event logs were collected using respective logging subsystems of the operating systems (Linux and Windows) installed on each host. The event logs were then forwarded to a central location. The network topology description was created manually.
**Data source location**	Masaryk University, Brno, Czech Republic
**Data accessibility**	Repository name: Zenodo Data identification number: 10.5281/zenodo.3746129 Direct URL to data: https://doi.org/10.5281/zenodo.3746129

## Value of the data

To the best of our knowledge, this is the first dataset providing network traffic traces and corresponding event logs from a complex cyber defense exercise where human operators deal with a number of attacks featuring recent vulnerabilities, applications, and systems.

• Since the *exercise network* was designed as a full-fledged digital twin of a fictitious organization, the data are equal to data generated in real enterprise networks. Yet, at the same time, indicators of multiple cybersecurity attacks can be found in the data, spanning a relatively short time interval.
• The main beneficiary group are cybersecurity experts and researchers that rely on primary security data in their work, e.g. in the areas of intrusion detection, traffic analysis, threat identification, and education and training.
• The data are normalized and stored in structured representation, and they are readily processable by common data analytics engines. All exercise devices were time-synchronized, which allows for sequence mining and correlation. The respective data payloads are in their raw form, ideal for subsequent processing.
• The dataset does not contain any personally identifiable data since all participants play a particular role according to the exercise scenario. All names and documents within the exercise are fictitious, crafted only for the exercise. This prevented distortion of important data features via anonymization process, without raising privacy concerns.

## 1 Data description

The dataset includes traffic flows and event logs from Linux and Windows machines captured and collected during a run of a Red Team/Blue Team cyber defense exercise held on March 19–20, 2019. The exercise format and lessons learned are presented in [1] in more detail. The *exercise network* was deployed in the KYPO Cyber Range Platform [2] and it was designed to be a full digital twin of a fictitious organization with all exercise hosts and network devices running common operating systems and applications, which can be found in modern organizations. The result of this are network and log data that are equal to data seen in the real world, without the need for anonymization, ready to be processed and analysed.

The dataset covers two distinct time intervals, which correspond to the official schedule of the exercise. The timestamps provided below are in the ISO 8601 date format.

• Day 1, March 19, 2019
  ○ Start: *2019-03-19T11:00:00.000000+01:00*
  ○ End: *2019-03-19T18:00:00.000000+01:00*
• Day 2, March 20, 2019
  ○ Start: *2019-03-20T08:00:00.000000+01:00*
  ○ End: *2019-03-20T15:30:00.000000+01:00*

The captured and collected data were normalized into three distinct event types and they are stored as structured JSON in order to be ready for direct processing and analysis. The data are sorted by a timestamp, which represents the time they were observed. Each event type includes a raw payload ready for further processing and analysis. The description of the respective event types and the corresponding data files follows.

• *cz.muni.csirt.IpfixEntry.tgz* – an archive of IPFIX traffic flows enriched with an additional payload of parsed application protocols in raw JSON. There are 469,113 events of this type. The captured traffic includes, for instance, these communication protocols: HTTP, DNS, DHCP, POP3, and SSH.
• cz.muni.csirt.SyslogEntry.tgz – an archive of Linux Syslog entries with the payload of corresponding text-based log messages. There are 6083,409 events of this type. The collected logs include, for instance, information from these daemons and applications: cron, smbd, sshd, usermod, useradd, and firewalld.
• *cz.muni.csirt.WinlogEntry.*tgz – an archive of Windows Event Log entries with the payload of original events in raw XML. There are 2901,154 events of this type. The collected logs include, for instance, information about these audited actions: user login, user logout, privilege escalation, and credentials validation.

Each archive listed above includes a directory of the same name with the following four files.

• *data.json.gz* – the actual data entries in a single gzipped JSON file.
• *dictionary.yml* – data dictionary for the entries.
• *schema.ddl* – data schema for Apache Spark analytics engine.
• *schema.jsch* – JSON schema for the entries.

Finally, the *exercise network* topology is described in a machine-readable NetJSON [3] format and it is a part of a set of auxiliary files archive – *auxiliary-material.tgz* – which includes the following.

• *global-gateway-config.json* – the network configuration of the global gateway in the NetJSON format.
• *global-gateway-routing.json* – the routing configuration of the global gateway in the NetJSON format.
• *redteam-attack-schedule.{csv,odt}* – the schedule of the Red Team attacks in CSV and ODT format. Source for Table 2.
• *redteam-reserved-ip-ranges.{csv,odt}* – the list of IP segments reserved for the Red Team in CSV and ODT format. Source for Table 1.

**Table 1:** IP address ranges reserved for the Red Team.

1.9.0.0/16	5.23.128.0/17	5.172.192.0/20	27.3.0.0/19
27.111.240.0/20	37.6.0.0/16	37.32.0.0/19	66.231.64.0/20
77.51.0.0/16	78.177.0.0/16	80.79.0.0/20	80.93.176.0/20
81.17.0.0/20	92.53.192.0/19	110.5.80.0/20	111.66.0.0/16
129.90.0.0/16	130.255.32.0/19	181.118.144.0/20	188.40.0.0/16
193.151.128.0/19	200.110.240.0/20	202.2.96.0/19	212.5.0.0/19
212.96.96.0/19	213.5.0.0/21	217.25.208.0/20	219.15.224.0/20
• *topology.{json,pdf,png}* – the topology of the complete Cyber Czech *exercise network* in the NetJSON, PDF and PNG format.
• *topology-small.{pdf,png}* – simplified topology in the PDF and PNG format. Source for Fig. 1.

**Fig. 1:**
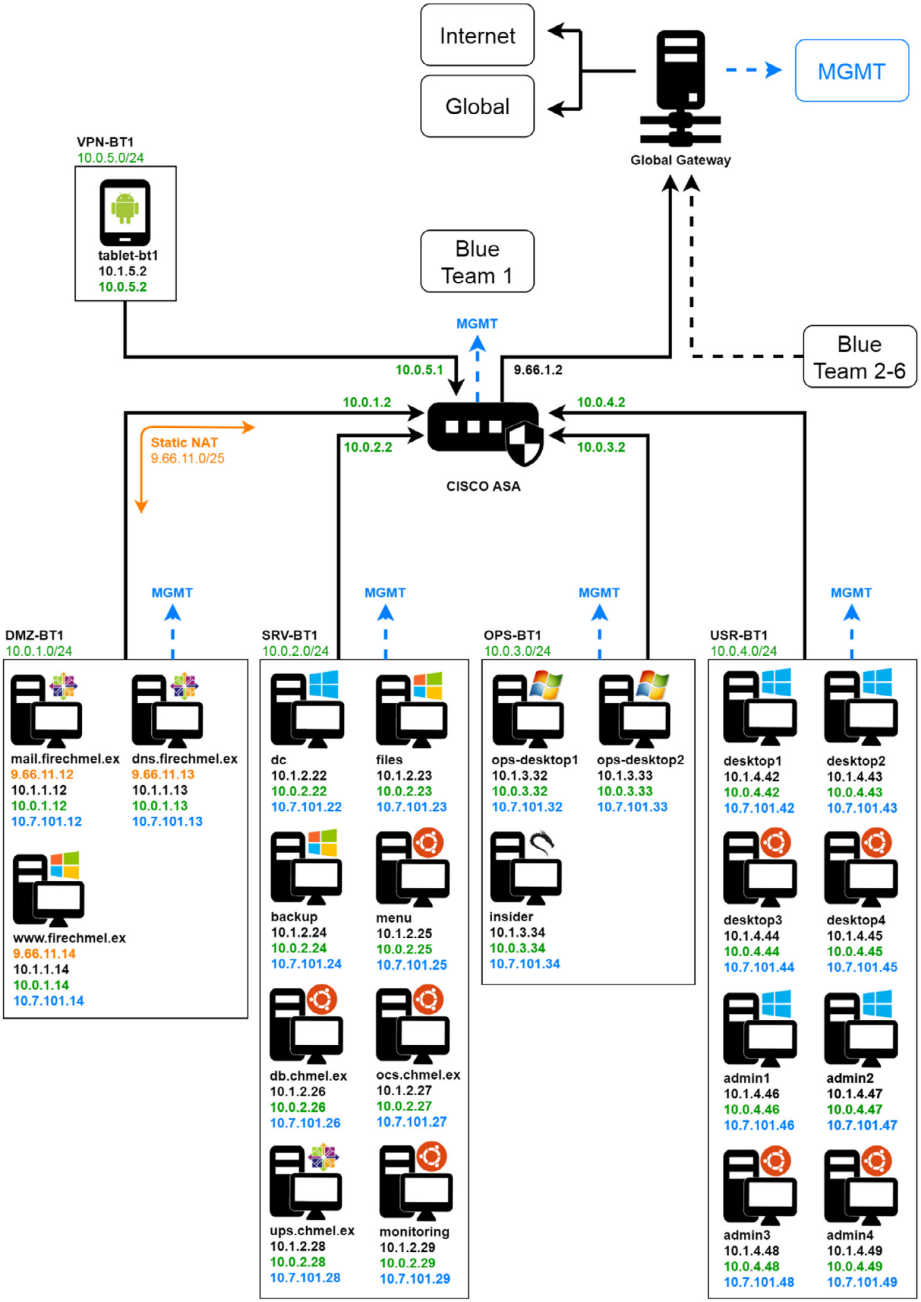
The simplified topology of the Cyber Czech exercise network. For the full topology see the topology.pdf in the auxiliary materials.

## 2 Experimental design, materials, and methods

The data were acquired during a two-day Cyber Czech exercise held on March 19–20, 2019 at Masaryk University, Brno, Czech Republic. The exercise was carried out on-site, i.e. on the premises of the university. The exercise network was built via KYPO cyber range platform developed and operated by Masaryk University. The main target group of the exercise were 20 learners coming from various bodies of public administration and critical information infrastructure of the Czech Republic invited by National Cyber and Information Security Agency (NCISA). All 20 learners provided their informed consent for the collection and publication of the dataset. The learners were grouped in five Blue Teams of four and protect emulated critical information infrastructure against attackers in Red Team. The goal of the exercise is to enable learners to experience cybersecurity incidents in a safe, isolated environment. The learners can practice both technical and soft skills for reacting to cybersecurity incidents, including prevention to future attacks, reporting to management, and informing other involved parties. The attacks were conducted by a group of experts named Red Team who have extensive knowledge about the *exercise network* and who have implanted selected vulnerabilities into exercise hosts. Members of Red Team were cybersecurity professionals and penetration testers from National Cyber and Information Security Agency (NCISA) and Computer Security Incident Response Team of Masaryk University (CSIRT-MU). The exercise also features a simulation of real users (employees) of the *exercise network* who fulfil their daily business tasks. The simulated users also came from NCISA and CSIRT-MU. The Blue Teams must not only secure their networks but also ensure the availability of defined network services and mission-critical applications to simulated users. The first day of the exercise is dedicated to familiarization of the Blue Teams with the *exercise network* since they do not know any technical details about the *exercise network* in advance. Blue Teams can also secure vulnerabilities they find during the familiarization.

The actual exercise takes place on the second day. It lasts 6 h without any break. The attackers follow the predefined scenario and schedule and conduct the attacks against the networks of Blue Teams. At the same time, simulated users interact with the network and fulfil their routine tasks. They may also contact the Blue Teams and ask for help if needed. Blue Teams must respond to their inquiries. Blue Teams are scored based on the availability of required network services and applications, response to attacks, and communication with simulated users.

All five Blue Teams were seated around five tables in a specialized physical room. Red Team and users simulating employees in the exercise were seated together in a different room in the same building. The only allowed communication channel between Blue Team, Red Team, and simulated employees was e-mail within the exercise network.

More details about the exercise and the used cyber range are provided in [1] and [2], respectively.

## 3 Network topology description

The exercise topology consists of two separate networks, the *exercise network* and the *management network*, that each serves a different purpose. The following section describes the two networks in greater detail. Their simplified topology is depicted in Fig. 1; however, it is recommended to see the more complete schema *topology.pdf* that is referred to in this section. It is available in the auxiliary materials.

The *exercise network* is a complex hierarchical network over which all the devices interact during the exercise. It is represented by the solid black lines in the topology schema. The *management network* is a support network with a flat star structure, designed to allow direct maintenance access to all devices before and during the exercise. It is represented by the blue dashed lines in the topology schema.

## 4 Exercise network

An illusion is kept from the view of the Blue Team members that the exercise comprises solely of their own *Blue Team network* and an insecure outside network – the Internet. This requires segmenting the exercise network into independent subnetworks. There are six *Blue Team networks* for each participating Blue Team (situated in the bottom half of the topology schema) and a single *global network* (situated in the upper half of the topology schema). The *global network* hosts exercise-wide services, like domain name resolution, mail services, and web services, and it also serves as an access point for the Red Team to the *exercise network*.

The single central point connecting the subnetworks of the *exercise network* together is the Global Gateway (depicted in the middle of the topology schema). The Global Gateway connects the *Blue Team networks* to the *global network* and facilitates connections outside of the *exercise network* to the Internet for all participants. Since many IP address ranges used in the exercise are public, the *exercise network* is isolated from the Internet by a NAT that translates the addresses of the participating devices before forwarding their communication outside. Besides the NAT to the Internet, the Global Gateway operates other NATs for the needs of both the Red Team and the Blue Teams. These are described in the Global Gateway Address Translation subsection.

Every device that actively participates in the exercise is connected to the *exercise network* by one or more physical interfaces. It follows that it needs an exercise-unique *global address* (black addresses in the topology schema). The addressing in the *exercise network* is a complex matter. It needs to provide the Red Team the freedom an attacker would have when attacking from any public IP address on the Internet and, at the same time, preserve the illusion that each one of the Blue Teams is the only one in the exercise. Aside from *global addresses*, devices might also be assigned other addresses where the exercise scenario requires it. The addresses used and their assignment process are different for the *global network* and *Blue Team networks* and are described in more detail in their corresponding subsections.

### 4.1 Global network

The *global network* represents a simulated “Internet” according to the exercise scenario. The simulated Internet is an indistinguishable part of the Internet for the Blue Teams, so from their view, the events of the scenario come from the Internet. The scenario is played through the devices connected in the global segment to all participating Blue Teams at once.

The devices in the *global network* divide into two groups – the service providers and the attackers. The service providers facilitate the legitimate DNS, e-mail, and web for the Blue Teams and legitimate virtual organizations that take part in the exercise, e.g. the Police and the local press agency. The Global Gateway, the central point providing routing and address translation for the exercise, also belongs to this network. The attackers serve as the access points of the Red Team to the *exercise network*. The attackers are not allowed to attack other devices in the *global network*.

The devices in the *global network* are connected as follows. The service providers have one network interface connected to the *global network*. The interface is assigned with a *global address* from the public range 4.122.55.0/24 to simulate a part of the Internet. The attackers have two connected network interfaces at their disposal and use the same range 4.122.55.0/24 for their *global addresses*. The second network interface of the attackers is used for anonymization purposes.

### 4.2 Red team anonymization

The Red Team is provided with anonymization based on jump segments. Its single purpose is to allow the attackers to continue attacking the *Blue Team network* even after their assigned IP address had been discovered and blacklisted by the Blue Team. The second network interface of the attackers may be switched dynamically to any IP address from a set of 28 predefined IP ranges during the exercise. If an IP address from these ranges becomes compromised, an attacker may immediately switch to a new one from the same or a different range. The ranges available to the Red Team are summarized in Table 1.

### 4.3 Blue team network

The *Blue Team networks* are separated from each other and no communication is possible between them. They represent an internal network and it is the sole responsibility of each corresponding Blue Team to protect it from the Red Team. Red Team attackers may consider any device in the *Blue Team network* as a valid target. The number and purpose of the connected devices, as well as further segmentation of the *Blue Team network*, are given by the exercise scenario. Its parameters are usually based on a well-known design commonly encountered in the real world.

All the six *Blue Team networks* in the exercise are identical. They consist of the same devices and use the same internal addressing. This design simplifies the building process and ensures that the networks of different Blue Teams are isolated. However, it also means that the Blue Team internal addressing is not unique in the scope of the *exercise network*. The *Blue Team internal addresses* (green addresses in the topology schema) need to be translated by a NAT when they communicate beyond the Global Gateway.

The addressing in the *Blue Team networks* uses private IP ranges to simulate an internal environment. All the Blue Team devices have one interface connected to the *exercise network* and it is assigned a *Blue Team internal address* from the range 10.0.0.0/16. The *Blue Team network* is further divided into five segments – demilitarized zone (DMZ), server (SRV), operations (OPS), user (USR), and virtual private network (VPN). The addressing in the DMZ and VPN segments differs slightly from the others, so it is explained further.

The DMZ segment contains services that should be accessible to the users in the *global network*. The services are accessible under the e*xercise public addresses* of the DMZ servers (orange addresses in the topology schema) from the *global network* only. The e*xercise public addresses* belong to the 9.66.XX.0/25 range, where X denotes the number of the Blue Team {1–6}. For example, the addresses for Blue Team 1 would be from the range 9.66.11.0/25. The addresses are not directly assigned to any network interface of the DMZ servers. Instead, they are translated to the server *Blue Team internal addresses* on the Cisco ASA appliance of the appropriate Blue Team.

The VPN segment is reserved for the mobile devices of the Blue teams. They are represented by Android tablets, that the Blue Teams may use during the exercise scenario. These devices connect to the *exercise network* through a wi-fi access point situated in the *global network*. However, they communicate exclusively through a VPN tunnelled connection directly to the Cisco ASA of the appropriate Blue Team.

### 4.4 Global gateway address translation

The Global Gateway is the central element interconnecting the separate *Blue Team networks* with the g*lobal network*. In total, the Global Gateway has seven interfaces connected to the *exercise network*. Six interfaces are reserved for the six *Blue Team networks* and one interface is connected to the *global network* and the Internet through a bridged interface. The Gateway performs network address translations to translate *Blue Team internal addresses* to *global addresses*, so that the same devices from the different *Blue Team networks* can be distinguished and addressed from the *global network.* The gateway also performs routing operations to allow the attackers to change their addresses dynamically during the exercise.

The Global Gateway uses static NAT to translate *Blue Team internal addresses* to *global addresses* to prevent conflicts when Blue Team devices communicate with the *global network*. To construct the *global address*, the Gateway uses the *Blue Team internal addresses* and the number of the corresponding Blue Team. When a Blue Team device communicates outside, a *global address* is chosen from the range 10.X.0.0/16, where X denotes the number of the Blue Team {1–6}. The process is reversed when a response is received, and the *global address* is translated back to the *Blue Team internal address*. For example, when the device *ops-desktop1* from the OPS segment of the Blue Team 2 communicates with the *global-web*, its address is translated from 10.0.3.32 to 10.2.3.32 (and back for the response) on the Global Gateway. The translation process uses iptables to mark packets and then routes them according to these marks.

The Red Team is provided with a set of IP address ranges so that the attackers can switch to a new one if any gets revealed during the exercise. The Global Gateway is configured to route these ranges to the *global network* where the attackers are situated, rather than outside of the *exercise network* to the Internet. This approach might disrupt operations of the Blue Teams if they tried to access an outside Internet server with an address from the Red Team ranges. However, the Red Team ranges must be public, so that the attackers do not stand out in the common network traffic. The Red Team ranges were specified with care to include only reserved and unused IP address ranges.

## 5 Management network

The *management network* of the Cyber Czech 2018 exercise is a support network serving two main purposes. The first purpose is to ensure a smooth run of the exercise. When a Blue Team reports a problem with any of its devices, the on-site support team uses the *management network* to access the device and check for the cause of the problem. The cause might be a misconfiguration or a successful Red Team attack, in which case only a hint to the Blue Team is provided, or an infrastructure error, which is promptly corrected. The second purpose is to transfer data critical to the exercise evaluation. During the exercise, data is collected from chosen Blue Team devices that allow measuring if the normal operation of the *Blue Team network* has been hampered. This data is first collected on the scoring server assigned to each Blue Team (*scoring-blue{1–6*} servers in the topology schema) and then stored and analysed on the central scoring server for the whole exercise *(central-scoring* server in the topology schema).

The abovementioned purposes could be served over the *exercise network* alone, without the need for a separate one. However, the *exercise network* is affected by Red Team attacks and Blue Team countermeasures during the exercise. When the scenario starts, the *exercise network* becomes too unstable and too unreliable for support purposes. The *management network* is separated from the *exercise network* to provide support even in the later and more destructive stages of the exercise. To keep the network isolated, no team present in the exercise is allowed to tamper with the *management network* interfaces and no scenario-dependent operations are allowed over this network.

All the devices in the exercise, excluding only the tablets, have one network interface connected to the *management network* and assigned with a *management address* (blue addresses in the topology schema) from the range 10.7.0.0/16*.* The central point of the star-shaped *management network* is the Global Gateway. There are also several devices that are connected to the *management network* only (*central-config, central-scoring, scoring-blue{1–6}*). These devices belong to the exercise backend and do not actively participate in the scenario.

## 6 Data capture

Three types of data were captured during the exercise: network traffic data, Linux syslog data, and Windows Event Log data. The network traffic data in the form of network flows represent the network view of the exercise. The log data category, on the other hand, provides the host-based view of the exercise through the events captured on every device connected to the *exercise network*, both from Linux and Windows machines. All the participating devices were time-synchronized to microseconds via a local NTP server to preserve relations between the events captured on different devices and network flows.

All three types of the data were collected into raw JSON and pre-processed using lossless data manipulation procedures, e.g. attribute renaming, object nesting, and attribute deduplication. Through this transformation process, the raw JSON data were normalized into structured JSON, i.e. described via a strict data schema.

### 6.1 Network traffic capture

The network traffic generated during the exercise was captured on the *global network* interface of the *Global Gateway*. This interface is the single point in the *exercise network*, where the communication between each Blue Team and the Red Team flows through, so all important transmissions during the exercise are captured. The network traffic was captured using tcpdump [4] in the form of PCAPs, exported into IPFIX flows [5] using a commercial Exporter [6], and finally converted into JSON via an open-source IPFIX Collector [7].

The devices in the data capture are identified by their *global addresses*, as the capture interface belongs to the *global network.* The g*lobal addresses* are coloured black in the topology schema. The attackers might also be identified by addresses from their reserved ranges (Table 1). The capture also contains other outlying addresses that do not belong to any device in the topology schema. This communication was not cut out of the dataset as we consider it a legitimate part of the set. The outliers might have been caused by misconfigurations carried out by Blue Teams and attacks of the Red Team, e.g. network scanning.

We explicitly mention what data can or cannot be expected in the network traffic capture. However, we do not provide an exhaustive list of all possibilities. The captured traffic **does** include interactions between the Red Team and the Blue Teams, and interactions of the Blue Teams with the Internet and the *global network*. It also includes the interaction of the users situated in the *global network* with the services in the *Blue Team networks*. The data capture **does not** include, most importantly, any communication that did not reach the Global Gateway. Namely, interactions of users situated within the *Blue Team networks* with their internal web and mail services. Interactions of the Red Team with the *global network* and outside Internet resources are also out of the scope of the capture.

### 6.2 Host-based event capture – Linux and windows logs

The devices in the event capture are identified by their *management addresses. Management addresses* are coloured blue in the topology schema. There are no outliers as the set of the monitored devices is clearly specified. The host-based data were captured both from Linux and Windows machines using their standard logging subsystems. Since the logging subsystems of these operating systems differ in nature and produce vastly different log entries, the resulting data are treated as two different data types.

Linux logs from each individual host include entries from various agents, e.g. services, applications, and OS kernel components, that were configured to use the syslog subsystem [8]. The log entries were then forwarded by a widely-used syslog daemon implementation – Rsyslog [9] – to a central syslog server via The Syslog Protocol [10]. The central server again used Rsyslog and it was configured to store the incoming log entries as raw JSON.

Windows logs from each individual host include entries from various agents, e.g. services, applications, and OS kernel components, that were configured to use the Windows Event Log subsystem [11]. The log entries were then forwarded by the Winlogbeat proprietary agent [12], used to ship WEL data over a network to a central server. The central server used Logstash [13] to collect the data and store them in raw JSON.

Contrary to the network traffic, the log data have been collected directly at the end-point devices, which were under control of the participants during the exercise. Any tampering with the configuration had been forbidden by the exercise rules to prevent unauthorized changes in the log generation and collection at these devices. However, any unsanctioned actions by the Blue Team members might have resulted in a loss of events for the device, until detected and corrected by the support team.

The captured events **do** include Linux and Windows log entries from the individual hosts produced many different applications and services ranging from DNS servers, through mail servers to monitoring services like Nagios. The captured events **do not** include, perhaps most notably, logs from the devices of the network infrastructure. Namely, the events originating in the Cisco ASA devices and also in the Global Gateway. Furthermore, the events from the Android tablets operated by the Blue Teams are not a part of the dataset.

## 7 Red team attack schedule

The Red Team had a schedule of attacks that were to be carried out against *Blue Team networks*. Table 2 provides an overview of the attack plan. However, the actual execution of particular attacks could have changed during the exercise as the Red Team tackled with different countermeasures taken by the Blue Teams. The Red Team members were also encouraged to be creative in hacking Blue Team resources.

**Table 2:** Schedule of attacks conducted within the exercise.

Exercise time [hh:mm]	Attack type	Importance	Affected network segments
00:00 – 00:30	Network reconnaissance	Low	DMZ
00:30 – 01:00	Denial of service	Low	DMZ
01:00 – 02:00	Web attacks	Medium	DMZ
01:30 – 02:00	Phishing	Medium	DMZ
02:00 – 02:40	Web attacks	High	DMZ
02:00 – 02:40	Ransomware	Medium	USR
02:30 – 03:00	Denial of service	Medium	DMZ
03:00 – 03:30	Sabotage of a mission-critical application	Medium	SRV
03:00 – 04:00	Data leakage	High	DMZ
03:00 – 04:00	Data leakage	High	USR
03:30 – 04:00	Sabotage of a mission-critical application	Very high	SRV
04:30 – 05:00	Sabotage of a mission-critical application	High	SRV
04:30 – 05:00	Denial of service	High	DMZ
05:00 – 05:30	Sabotage of a mission-critical application	Very high	SRV
05:30 – 06:00	Sabotage of non-critical parts of the infrastructure	Very high	All segments
05:50 – 06:00	Sabotage of a mission-critical application	Very high	SRV

## Declaration of Competing Interest

The authors declare that they have no known competing financial interests or personal relationships which have, or could be perceived to have, influenced the work reported in this article.

## Appendix Supplementary materials

Image, application 1
